# Trade Impacts on Embodied Carbon Emissions—Evidence from the Bilateral Trade between China and Germany

**DOI:** 10.3390/ijerph17145076

**Published:** 2020-07-14

**Authors:** Jiajia Li, Abbas Ali Chandio, Yucong Liu

**Affiliations:** College of Economics, Sichuan Agricultural University, Chengdu 611130, China; jiajia_li108@hotmail.com (J.L.); okaysummer@163.com (Y.L.)

**Keywords:** bilateral trade, embodied carbon emissions, CO_2_ transfer, China, Germany

## Abstract

This article attempts to investigate the impacts of bilateral trade on the environment by estimating the embodied carbon emissions between China and Germany over the period 1999–2018. The above impacts are broadly explored in the literature both under the framework of theoretical and empirical analysis. However, there exist fewer empirical studies exploring the nonlinear relationship between trade volumes and carbon emissions between a well-developed and emerging economies. By applying the multiregional input-output (MRIO) model, this article aims to reveal the impacts of trade on the environment in the case of China–Germany. Specifically, trade amounts between China and Germany rank high with a similarly increasing trend and both of them are large net exporting countries. However, China experienced much larger carbon emissions embodied in its exports to Germany. Despite potential concerns on the carbon leakage issue of China from Germany, we find that the bilateral trades fit an inverse U-shape in the embodied carbon emissions, which suggests that the trade between the two countries can finally reduce carbon intensity without obstructing economic development particularly in the long-term. This paper guides policy-makers to quantify the issue of CO_2_ transfer among bilateral trades in order to achieve the target of trading sustainability.

## 1. Introduction

Presently, developing, as well developed, countries around the world are ambitious to mitigate the impacts of greenhouse gas (GHG) emissions under the circumstances of globalization and climate change. On one hand, carbon emissions are regarded as the most significant contributor to GHG emissions. On the other hand, international trade is a vital issue in the globalization process. Therefore, environmental consequences originating from international trade cause growing concerns by scholars and policy-makers in recent years [1].

The impact of international trade on the environment has been explored in depth in the previous studies (see for example, Rauscher [2]; Zhang et al. [3]; Halicioglu and Ketenci [4]). Similarly, a couple of studies explore the interactions between trade and carbon emissions, but the environmental consequences of trade are not consistent. The classic theories regarding trade creating pollution suggest that developed countries tend to migrate the dirty industries to less developed countries [5]. On the contrary, some other studies proposed that international trade motivates countries to update technology [6], which is beneficial for the environment in the long-term. Grossman and Krueger [7] enhanced the theory of the associations between trade and the environment by decomposing the trade impacts on pollutions into scale, composition and technique effects. Notably, the economic theory of “environmental externality” further verifies the uncertainty of the effects of trade on the environment [8]. Hence, exploring the unobserved linkages between trade and carbon emissions is significant especially for specific situations and for different data-bases.

Regarding the related empirical studies, the previous literatures mainly explained the role of trade openness in carbon emissions, and some studies found that trade liberalization increases CO_2_ emissions especially in developing countries, for instance China [3]. However, there are less studies establishing the impacts of trade amounts on the embodied CO_2_ emissions. Comparing with trade openness, trade volumes are more accurate and direct in understanding the embodied carbon emissions generated from the trade. A couple of other studies focused on direct carbon emissions generated from the energy sector only (see for example, Jin et al. [9]). Thus, indirect carbon emissions are largely ignored. Therefore, it is less accurate to investigate the issue of CO_2_ transfer in international trade. Following the logic above, this article aims to examine the impacts of bilateral trade volumes on embodied carbon emissions by applying the latest time series data.

Examining the environmental consequences generated from bilateral trades between developing and well-developed countries is a crucial research direction, as the potential carbon leakage is more likely to happen among countries with differences in environmental regulations and/or economic development [9,10]. Individually, China has become the largest carbon emission emitter in the world since 2007, and its active participation in the World Trade Organization (WTO) could offer thoughtful policy implications in the trade and environment [11]. Several studies tend to investigate the bilateral trade relationship of China and some other well-developed countries, such as US [12], Japan [13] and Australia [14]. However, only a few studies focused on the bilateral trade between China and Germany, and its impacts on the embodied carbon emissions are also comparatively rare.

**Figure 1:**
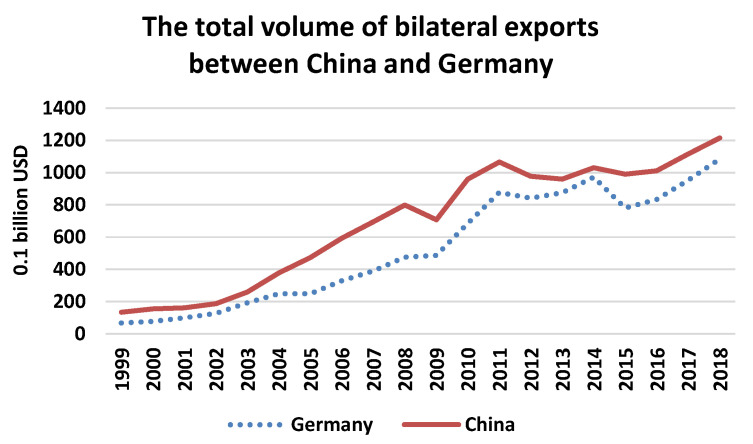
The trends of total bilateral exports between China and Germany from 1999 to 2018. Note: In the bottom of the graph, “Germany” stands for the export volume of Germany to China, and “China” is the export volume of China to Germany.

China and Germany are both playing significant roles in international trade, and they ranked the second and the third in global trade in 2019, respectively. In addition, both China and Germany are export-oriented countries with potential pressure on emissions reduction [15]. In this regard, investigating the bilateral trade volumes embodied in carbon emissions is important in understanding the emissions gap for countries with the problem of CO2 transfer in trade. China persistently takes the leading trading partner of Germany, and Germany is also the top trading partner in the European Union (EU) for China, which accounted for more than 40% of the EU’s total exports to China in recent years [16]. In 2018, the bilateral trading values between the two countries reached 1.8 trillion USD. The trends of bilateral export from 1999 to 2018 are shown in Figure 1. The graph illustrates the bilateral trade between China and Germany kept growing rapidly from two decades ago, notwithstanding China consistently exceeds net exports to Germany. The consistent net exports create a potential carbon leakage risk to China. However, it is too simple to conclude that developing countries like China have created more pollution due to gross export growth, as the long-run carbon emissions generated from exports could also become environmental-friendly with updated technology and enhanced energy efficiency which lead to greener productions in various industries.

Following the above analysis, this study concentrates on the bilateral trade between China, which represents a developing economic entity, and Germany, a well-developed country, and examines the impacts of bilateral total exports on embodied carbon emissions from 1999 to 2018. The relative theories address the issue that the impacts of trade on the environment are unobvious and uncertain, and our study has two main research targets: first, we employ the Multiregional Input-Output (MRIO) analysis to calculate the embodied carbon emissions generated from the bilateral trades, and identify carbon leakage behind the bilateral trade between China and Germany, respectively. Second, we apply the autoregressive distributed lag (ARDL) approach, fully modified ordinary least squares (FMOLS), canonical cointegration regression (CCR) techniques, and some other robustness tests to establish the association between trade volumes and its embodied carbon emissions. The empirical processes are important in order to further investigate the series of theories regarding the linkage of trade and the environment.

This article fills the gap by comparing, in depth, the bilateral trade volumes and their embodied carbon emissions for China and Germany, and further explores the nonlinear relationship between trade volumes and the embodied carbon emissions. Specifically, China and Germany have comparable trade amounts on bilateral exports but could turn out totally different carbon emissions. In addition, although larger carbon emissions are in line with larger export volumes, the relationship between the trade volume and its generated carbon emissions is worth exploring to determine whether emissions will finally reduce with higher production efficiency and economies of scale. Furthermore, this study covers 20 years of data which are relatively longer in period of time with the latest records.

The remainder of the paper is organized as follows: Section 2 outlines and summarizes literature. Section 3 shows the details of data use in this study and describes the MRIO method which is applied for calculating carbon emissions. Afterwards, this section shows the econometric models for regression analysis. The results and the robustness checks are reported in Section 4. Finally, Section 5 concludes and discusses the findings of this article.

## 2. Literature Review

There exist, primarily, three different views of theoretical background in understanding the impacts of trade on the environment. The theories of “Pollution Haven Hypothesis” [5] and “Race to the Bottom Hypothesis” [17] revealed that expanding trade could result in a polluted environment, and the above theories were also proved in empirical studies (see for example, Ederington and Minier [18]; Dean et al. [19]). Meanwhile, the issues of carbon leakage and CO_2_ transfer happen among trading partners; for instance, relocating productions to countries with less stricter emission reduction standards [5,20] and transferring carbon emissions from developed countries to developing ones [21]. The above theories and phenomena described the adverse effects of international trade on the environment. However, there exist some other theories that were more positive toward the impacts above. A couple of studies concluded that trade can enhance the environment, see for example, Antweiler et al. [6]; Dogan et al. [22]. Similarly, Birdsall and Wheeler [23] investigated that free trade decreases pollution by stimulating clean production technologies. There exists a third view of studies supported the complex associations between trade and the environment [24,25]. The related literature suggested the linkage between trade and environment are nonlinear and various in different trading stages [26,27]. For example, the “Environmental Kuznets Curve Hypothesis” was originally proposed to address the nonlinear impacts of economic growth on environment [28]. This theory describes that the environment becomes worse at the beginning stage with the growth of economy, but afterwards, the environment recovers with the continually prosperous economy.

Afterwards, research linking trade and carbon emissions had developed into various channels. Existing studies explored the impacts of trades on carbon emissions from different perspectives, see for example Andersson proved that institutional factors were important in reducing carbon emissions for trades among developing and developed countries [29]. This research provided a timely direction on not just obstructing trade expansion but exploring other ways, such as institutions and legal systems, in carbon emissions mitigation. Fu and Zhang analyzed carbon emissions for Chinese manufacturing sectors, and found that trades reduce carbon emissions for low carbon industries only [30]. This study also argued that estimating embodied carbons in trades is one of the most direct ways to address the effect of trade on carbon leakage.

Regarding to the methods of examining the environmental consequences of the trade volume on embodied carbon emissions in empirical research, the previous studies applied the ARDL method and FMOLS approach to establish trades and carbon emissions nexus (see for example, Nazir [31]; Khan et al. [32]; Jebli and Youssef [33]). Previous literature related to international trades between China and EU is well documented in various sectors (see for example, Li et al. [34]; Fang and Shakur [35]). Studies which focused on the trade between China and Germany mainly compare the bilateral trade volume itself, while others analyzed the reasons of the key contributors in stimulating the bilateral trades [16,36]. Guo et al. [37] summarized China’s exports to Germany developed since China’s access to WTO in 2001. Recently, Wang et al. [15] researched the carbon emissions embodied in trade for China and Germany, and discovered increasing volumes of carbon emissions between the two countries from 1995 to 2009. However, studies exploring the environmental consequences of trade between China and Germany for the latest data are rare.

Carbon emission is derived from the definition of “embodied energy” proposed by the International Federation of Institutes for Advanced Study (IFIAS) in 1974. There are mainly two popular input-output methods to calculate the embodied carbon emissions for trade. One is the single region input-output (SRIO) model, and another is called the multiregional input-output (MRIO) model. SRIO is common in researching the emissions embodied in trade, and treats all other countries and regions as a single entity [38]. SRIO is divided into a competitive input-output (IO) model and a non-competitive IO model. The competitive IO model does not distinguish between imports in intermediates and final-use products. On the contrary, the non-competitive IO model distinguishes import products from domestic products. Comparatively, the process of obtaining the carbon emissions through MRIO can go beyond the scope of the national economy. MRIO contains technical heterogeneities in various countries, and classifies imports into the intermediate input goods and final productions [15,39]. Our work employs the method of MRIO, as the empirical models include the technology term, and based on the literature reviews above, the MRIO method is more appropriate and accurate in our case comparing with the SRIO.

In summary, the aforementioned studies have deeply explored the impacts of trades on carbon emission with the support of various theories, and the existing literatures show that different measurements or data could turn out different results. However, investigating the impacts of bilateral trade amounts on the environment by calculating embodied carbon emissions between China and Germany is significant but neglected. This paper aims to fill the gap.

## 3. Data and Methodology

This section explains the data used in this study, and also describes the input-output method based on the MRIO analysis for calculating embodied carbon emissions. Afterwards, we will report the descriptive statistics of the main variables and compare the embodied carbon emissions generated from bilateral exports in total volumes between China and Germany from 1999 to 2018. Finally, this section will show the econometric models and explain the reasons of applying them.

### 3.1. Data

Data used in this study contains multiregional input-output tables of China and Germany which were collected from the World Input Output Data (WIOD (Data access from: http://www.wiod.org/database/wiots16)) from 1999 to 2014. The bilateral trade of the total volumes of China and Germany were extracted from UNCOMTRADE (Data access from: https://comtrade.un.org/). Energy consumptions by different types for China were gathered from the Statistic Yearbook of China (Data access from: http://www.stats.gov.cn/tjsj/ndsj/), and we will follow the input-output method to calculate coefficients of CO_2_ emissions for different sorts of energy. Similar data for Germany can be found in WIOD from the table of CO_2_ emissions (Data access from: http://www.wiod.org/database/eas13). Other control variables include urbanization rates and R&D intensities of the two countries which were collected from the World Bank (Data access from: https://data.worldbank.org/). We also control for a dummy variable of China’s participation in the WTO (in the year 2001) because, since then, the trades between China and the world have been more tightly connected.

Due to the input-output tables not available since 2015, we follow the approach of Ma et al. [40], and regard the year 2014 as the basic year (this article applies 2014 as the basic year because, currently, the latest input-output table is available for the year 2014 and, thus, more accurate estimations can be obtained for 2015–2018 compared with applying the previous input-output tables. Similarly, some other researchers employed the same method to obtain the carbon emission coefficients [40]) to estimate carbon emissions for the following years (2015–2018). The International Energy Agency (IEA) (Data access from: https://www.iea.org/) offers coefficients of total carbon emissions for each year, and we calculate the ratio of those coefficients for the targeting years to the coefficient of the basic year (C_t_/C_2014_), respectively, and then we obtain one carbon emission index for each year to further apply those indices in obtaining the embodied carbon emission coefficients.

### 3.2. Calculating Carbon Emissions

Firstly, this study calculates the direct carbon emission coefficients for each industry in China and Germany, respectively. According to the Statistic Yearbook of China, energy consumptions are divided into nine types. However, China’s electricity is mainly generated from coal and natural gas and, hence, we consider eight energy types including coal, gasoline, fuel oil, kerosene, crude oil, diesel oil, coke, and natural gas, respectively. The direct carbon emissions coefficients are calculated by transferring energy consumption to carbon emissions [41]. The above coefficients are available on WIOD for Germany. Afterwards, we can obtain the direct carbon emission coefficients matrix *R_j,i,t_*, where *j* is China or Germany, *i* represents industry, and *t* is year. *R_j,i,t_* is the direct carbon emissions of producing one unit of the production for industry *i*.

Secondly, based on input-output analysis, we apply multiregional input-output tables in order to establish the embodied carbon emissions *C_j,i,t_* (see Equation (1)) [21,42,43].

(1) $$C_{j,i,t}=R_{j,i,t}\times {\left(I-A_{j,i,t}{}^{D}\right)}^{-1}\times Y_{j,i,t}$$

where $R_{j,i,t}$ is the direct carbon emission coefficients matrix which has been calculated in the first step; $Y_{j,i,t}$ is the bilateral exports volumes; ${\left(I-A_{j,i,t}{}^{D}\right)}^{-1}$ represents that producing one unit of final goods for industry *i* will consume the amounts of goods in other industries, which is a style of Leontief inversed matrix after steps of derivations. Based on the previous analysis, $R_{j,i,t}\times {\left(I-A_{j,i,t}{}^{D}\right)}^{-1}$ represents the embodied carbon emissions of producing one unit of the good for industry *i*, country *j*, in year *t.*

Finally, $C_{j,t}$ is the total embodied carbon emissions generated from bilateral trades by adding the embodied carbon emissions of 14 industries together (see Equation (2)).

(2) $$C_{j,t}=\overset{14}{\underset{i=1}{\sum }}C_{j,i,t}$$

### 3.3. Descriptive Statistics and Correlation Analysis

This subsection lists the descriptive statistics of the variables in our econometric models, including the total embodied carbon emissions, bilateral trading amounts and their squared terms, urbanization rates, and R&D intensities for China and Germany, respectively. Additionally, we control a dummy variable of China’s participation in the WTO. The reasons of including the above variables are as follows: based on the existing theories [24,25], the nonlinear relationship between trade volumes and the embodied carbon emissions suggests adding a squared term of the logarithm for the bilateral trade amount. The economic thought behind the nonlinear model is that the carbon emissions could grow with the trade volumes first, but in the long-term the advantages in bilateral trades are becoming significant, the carbon emissions generated from the trade are shrinking with time. In addition, China’s accession to the WTO, the R&D intensity, and urbanization rates could also have effects on carbon emissions, as China’s participation in the WTO stimulates the international trade, which could lead to larger amounts of embodied carbon emissions [15]. Similarly, following the literature of Ge et al. [44], higher levels of urbanization increase the demand of production and consumption, which could generate more carbon emissions. On the contrary, the R&D intensity represents the technology of a country in a certain period of time [45]. Based on the previous studies [9], higher levels of technology make energy more efficient in the production process and, hence, the embodied carbon emissions tend to decline with the updates of the technology.

Thereafter, we compare the bilateral embodied carbon emissions with bilateral trade volumes. Table 1 presents the definitions of the main variables in the regression models. Table 2 reports the summary of descriptive statistics results for China. The results of the Jarque–Bera test suggest that variables are normally distributed, due to large *p*-values.

Table 3 shows the estimated results of correlation analysis, indicating that export volumes, WTO participating, R&D intensity, and urbanization are significantly positively associated with the embodied carbon emissions for China’s exports to Germany.

Similarly, Table 4 shows the summary of descriptive statistics results for Germany. The Jarque-Bera test also proves that the main variables of Germany are normally distributed, as large numbers of *p*-values show the normally distributed of the data. Table 5 reports the estimated results of correlation analysis, indicating that the main variables are significantly positively correlated with embodied carbon emissions for the total export amounts from China to Germany.

After comparing the mean values of the main variables in Table 2 and Table 4, we can figure out that the average embodied carbon emissions for China’s exporting to Germany are much larger than the emissions for Germany’s exports to China. The above phenomenon suggests China experienced larger carbon emissions, while the trading goods/services were consumed by Germany. In addition, the above differences of carbon emissions are much more significant compared with the differences of the bilateral trade volumes. It also shows that Germany has higher technology levels and urbanization levels compared with China.

Table 6 gives more details on the bilateral trade volumes and the embodied carbon emissions generated from the trades over the period 1999–2018. The embodied carbon emissions are calculated according to Equations (1) and (2). Regarding to the accuracy of calculating results for embodied carbon emissions, previous studies are taken for comparison. Comparing with the previous literatures, we obtain similar embodied carbon emissions coefficients with Ma et al. from 2000 to 2007 [40]. In addition, based on the embodied carbon emissions coefficients for China, we obtain the same results with Fu and Zhang by sorting industries into the high carbon group and the low carbon group [30].

**Figure 2:**
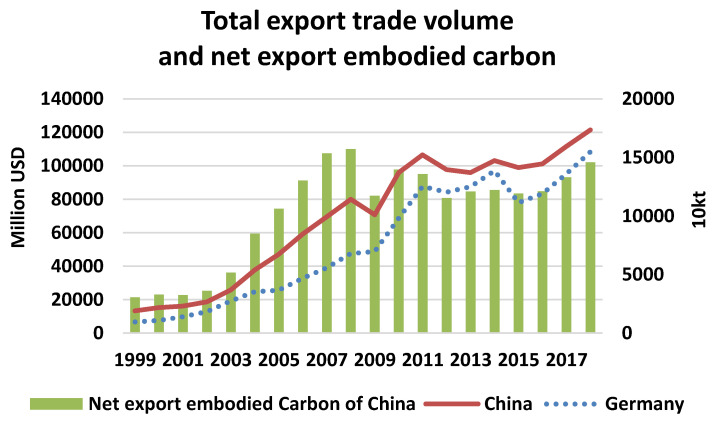
Comparing bilateral trades and the net exporting carbon emissions from China. Note: In the bottom of the graph, “Germany” stands for the export volume of Germany to China, and “China” is the export volume of China to Germany.

From Table 6, particularly, the net bilateral exports embodied in carbon emissions from China to Germany are 145,836 kt in 2018. It is important to know during the same period, carbon emissions generating from Germany’s exporting to China grew 10 times (from 1050 kt in 1999 to 10,954 kt in 2018), and the net exporting carbon emissions from China to Germany grew from 30,391 kt in 1999 to 145,836 kt in 2018. In general, with rapidly increasing trades between China and Germany, carbon emissions of both countries are rising with time. Table 6 and Figure 2 combined to illustrate that the net exporting embodied in carbon emissions from China to Germany peaked in 2008, afterwards it shows a trend of decreasing. Although there exists a stable growing trend in recent years, the embodied carbon emissions are mainly due to the net bilateral export growth.

### 3.4. Econometric Models

After calculating the embodied carbon emissions generated from trades between China and Germany, this article aims to explore the environmental consequence of the trade volume and, therefore, this article adds a squared term of the trade volume in to further examine the invers U-shape between trade volume and the embodied carbon emissions generated from the bilateral trades. Equations (3) and (4) exhibit the impacts of total bilateral trades on embodied carbon emissions for China and Germany, respectively.

(3) $$LNCO_{2C,t}=\alpha_{0}+\alpha_{1}LNTRA_{C,t}+\alpha_{2}LNTRA_{C,t}^{2}+\alpha_{3}WTO_{t}+\alpha_{4}TECH_{C,t}+\alpha_{5}LNURB_{C,t}+\mu_{C,t}$$

(4) $$LNCO_{2G,t}=\alpha_{0}+\alpha_{1}LNTRA_{G,t}+\alpha_{2}LNTRA_{G,t}^{2}+\alpha_{3}WTO_{t}+\alpha_{4}TECH_{G,t}+\alpha_{5}LNURB_{G,t}+\varepsilon_{G,t}$$

We mainly employ two models for empirical research, Equation (3) (Model 1) estimates the causal relationship between the bilateral trades from China to Germany and the estimated carbon emissions generated from those exports. Similarly, Equation (4) (Model 2) examines the same relationship from Germany to China, where $LNCO_{2C,t}$ is the logarithm term for the total embodied carbon emissions generated by the trade from China to Germany. $LNCO_{2G,t}$ represents the logarithm term of the carbon emissions which Germany’s exporting amounts to China. LNTRA is the total export volume for China or Germany, in year t. The square term of LNTAR is added in to examine the inverse U-shape of trade amounts on embodied carbon emissions. $WTO_{t}$ is a dummy variable that represents China’s accession to the WTO. In addition, the R&D intensity and the urbanization rate are also included in the two models above. $\mu_{C,t}$ and $\varepsilon_{G,t}$ are the residential terms for the two models, respectively. We apply the ARDL, FMOLS, CCR models to examine the linkage between the bilateral trades and the carbon emissions. Additionally, we involve in a couple of robustness tests, such as the bounds testing, cumulative sum (CUSUM), and cumulative sum of squares (CUSUMSQ) to test the stability of the ARDL models.

## 4. Results

### 4.1. Pre-Estimating Test

Firstly, we apply the Dickey–Fuller generalized least squares (DF-GLS) test and the Phillips–Perron (PP) test to check the stationary of the variables for the above two Models (see Equations (3) and (4), respectively). Table 7 reports the unit root tests results for Model 1—Bilateral trade from China to Germany. Similarly, Table 8 shows the unit root tests results for Model 2—Bilateral trade from Germany to China.

The estimated results of both the DF-GLS and the PP unit root tests are reported in Table 7 and Table 8, indicating that total embodied carbon emissions, total export volume, square of the total export volume, participation in the WTO, R&D intensity, and urbanization are integrated at first difference. These outcomes of both unit root tests confirm that we can use the ARDL model to explore the long-term relationship between the variables.

### 4.2. ARDL Bound Testing Approach Results

In order to further investigate the long-run relationships among the variables, this study applies the ARDL bound testing approach. Shahbaz and Sinha pointed out the ARDL bounds testing method is the most appropriate way to examine an inverse U-shape relationship for time series data in particular [46]. The estimated results of the ARDL bounds testing approach show that the computed F-statistics 10.62 and 3.90 are greater than the upper bound values at 1% and 10% significance levels (see Table 9). This means there exists a long-run association among the variables.

### 4.3. Estimated Results for China’s Total Exports to Germany

We first report the empirical results for China’s exporting to Germany. The estimated long-and short-run results for the effects of total exports volume, total exports volume of square, participation into WTO, R&D intensity, and urbanization on embodied carbon emissions are demonstrated in Table 10. The most important variables in the estimation are the total export volume and its squared term. The coefficient of the total export volume is positive and highly significant at 1% in the long-run, and the coefficient for the squared terms of the total exports from China to Germany is negative and highly significant at 1% in the long-run as well. These above results imply an inverse U-shaped relationship between the trade volume and its carbon emissions for the long-run period. In particular, when the bilateral exports from China to Germany are expiring at a relatively lower level, a 1% increase in total exports volume from China to Germany will lead to an increase in the embodied carbon emissions by 22.2%. With the growth of the bilateral trade amounts, these results suggest a confirmation of China’s total exports to Germany fit the inverse U-shape, which means carbon emissions embodied in trade will go down in the long-term.

Furthermore, Table 10 shows that the coefficient of technology is negative and highly significant at 1% in the long-run. This means that a 1% increase in technology will lead to a decrease in embodied carbon emissions by around 1.2% in the long-run. Therefore, technology updates are essential for restraining carbon emissions in trades. The coefficients of the WTO and urbanization are positively significant, which imply that a 1% increase in participation in the WTO and urbanization will increase embodied carbon emissions by 0.1% and 1.6% in the long-run, respectively.

**Figure 3:**
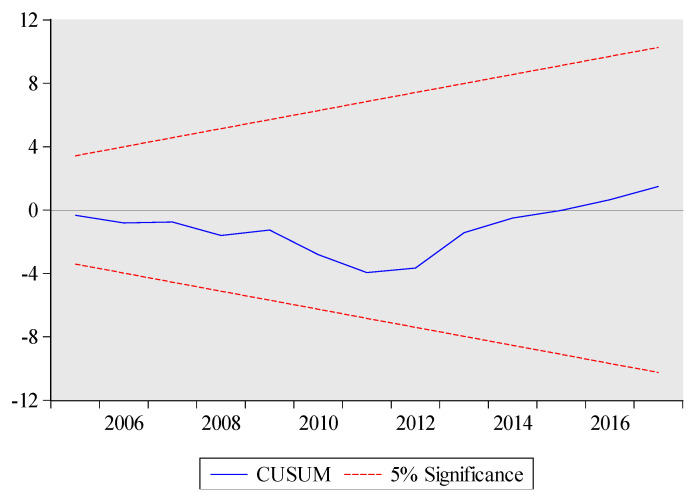
CUSUM test for Model 1. The plot of the cumulative sum of recursive residuals. Note: The red dashed lines represent the critical bounds at the five percent significance level.

**Figure 4:**
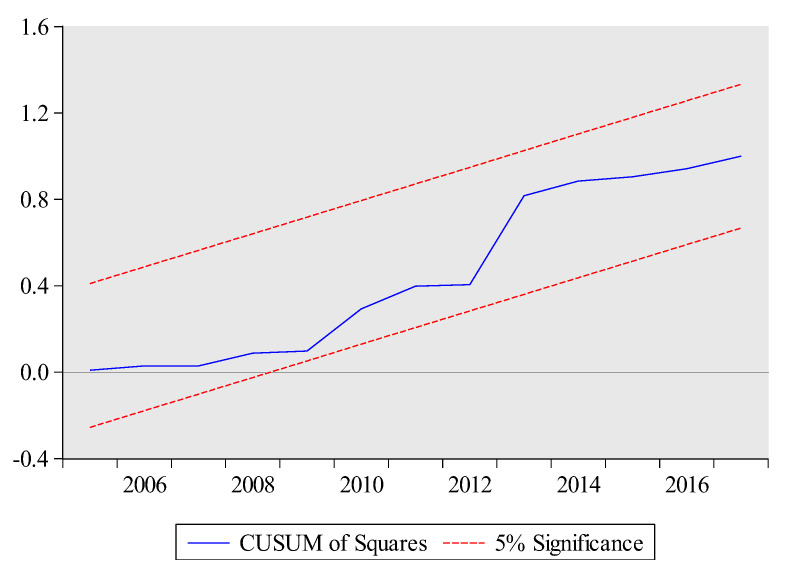
CUSUMSQ test for Model 1. The plot of the cumulative sum of squares of the recursive residuals. Note: The red dashed lines represent the critical bounds at the five percent significance level.

The estimated short-run results are also reported at the bottom of Table 10. The lagged coefficients of total exports volume and its squared term are significantly positive and negative, respectively. These results suggest that there is an inverse U-shaped relationship between total exports volume and embodied carbon emissions for short-term as well. The short-run coefficient of technology is negative and highly significant at 1%, which implies that a 1% increase in technology will lead to decrease embodied carbon emissions by 1.3%. Likewise, the lagged short-run coefficient of urbanization is positive significant at 1%, which implies that a 1% increase in urbanization increase embodied carbon emissions in the current period. The ARDL model has passed all diagnostic tests which suggest model 1 (see Equation (3)) is stable. In particular, the graphs of CUSUM and CUSUMSQ also prove the stability of the ARDL results (see Figure 3 and Figure 4). The high value of R-squared demonstrates that 0.99% variation in embodied carbon emissions is due to all independent variables.

For robustness checks of the above analysis, we apply the FMOLS and the CCR techniques. The estimated results of both approaches are reported in Table 11. The results exhibited that total export volume has a significantly positive effect on embodied carbon emissions while a squared term of total export volume has a significantly negative effect in both FMOLS and CCR estimations. Therefore, these findings suggest that there is an inverse U-shaped association between the total export volume and the embodied carbon emissions for China’s exports to Germany. The estimated outcomes of the FMOLS and the CCR methods support to the outcomes of the ARDL approach and. Overall, our results are robust.

### 4.4. Estimated Results for Germany’s Total Exports to China

**Figure 5:**
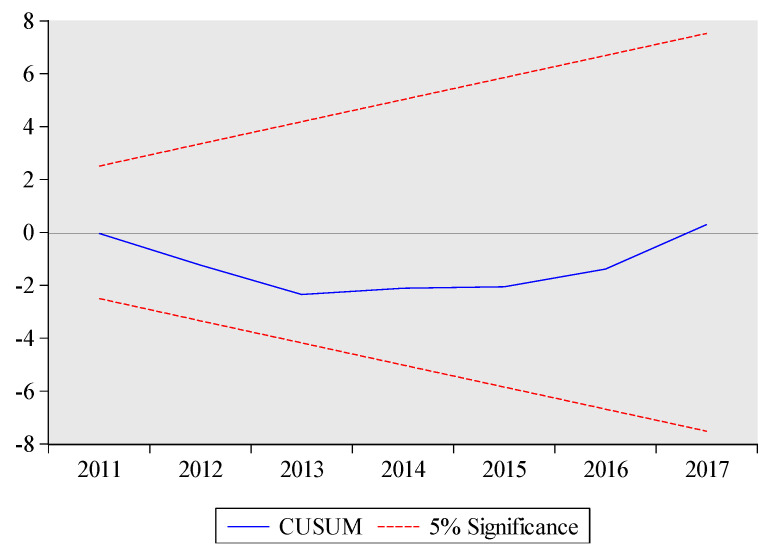
CUSUM test for Model 2. The plot of the cumulative sum of recursive residuals. Note: The red dashed lines represent the critical bounds at the five percent significance level.

**Figure 6:**
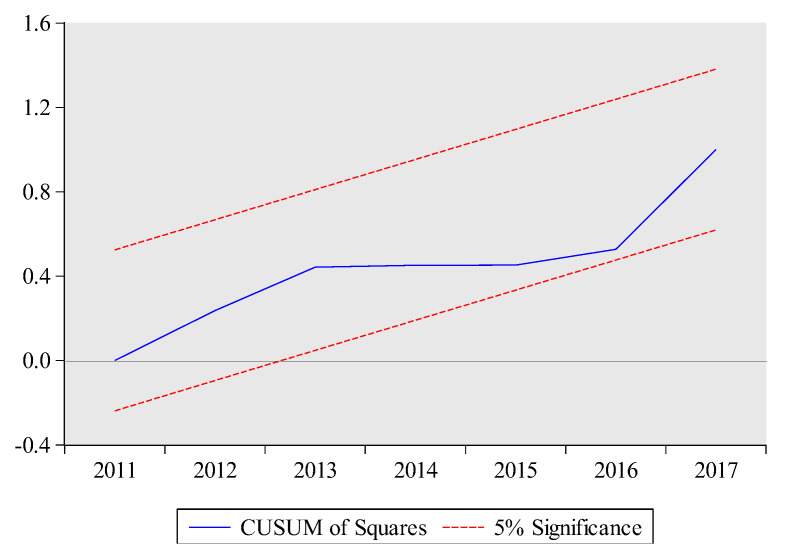
CUSUMSQ test for Model 2. The plot of the cumulative sum of squares of the recursive residuals. Note: The red dashed lines represent the critical bounds at the five percent significance level.

The estimated long-run and short-run results for Germany’s exports to China (Model 2) are presented in Table 12. The coefficient of the total exports volume is positive but insignificant in both long-run and short-run. Likewise, coefficient of a squared term of total exports volume is negative but insignificant in both the long-run and short-run. These results suggest that Germany’s total exports to China do not significantly fit declining carbon emissions with higher levels of trade amounts for the ARDL model as is the case of China’s total exports to Germany. The ARDL model (see Equation (4) is stable (see Figure 5 and Figure 6) and has passed various diagnostic tests (see the bottom part of Table 12).

In this study, we further apply the FMOLS and the CCR techniques to check the robustness of our previous results. Table 13 reports the estimated results of both the FMOLS and CCR estimations. The coefficient of total export volume has statistically significant positive impact on embodied carbon emissions. This result implies that a 1% increase in total export volume from Germany to China will lead to an increase in embodied carbon emissions by 4.0% and 5.0% based on the FMOLS and the CCR estimations, respectively. Likewise, the estimated coefficient of a squared term of total export volume from Germany to China has statistically significant negative impact on embodied carbon emissions. Therefore, these estimated outcomes suggest that Germany’s total exports to China fit the inverse U-shape based on the FMOLS and the CCR estimations.

## 5. Conclusion and Discussion

China and Germany have participated in a couple of international climate treaties regarding the mitigation of the impacts of climate change, such as the Copenhagen Accord and the Paris Agreement, and both of the countries are net exporting entities that bear more pressures to achieve the carbon reduction targets. Governments are taking actions to control carbon emissions through different aspects of activities. Trade is the most significant aspect to generate and/or transfer carbon emissions among trading partners and, hence, understanding the causal relationship between trades and embodied carbon emissions is essential to investigate the environmental consequences and guide further trading policies for China and Germany who possess tight cooperation in trades.

This paper explores the bilateral trade impacts between China and Germany on embodied carbon emissions from 1999 to 2018. By doing so, we employ the bilateral export volumes and calculate the embodied carbon emissions in revealing the associations between trade and the environment for the case of China–Germany.

Specifically, by applying the MRIO method in the process of determining embodied carbon emissions, this article reveals that there exists a gap in which the net carbon emissions embodied in the bilateral trade are always positive for China in the last two decades. Notwithstanding the gap of net carbon emissions between China and Germany has become stable since 2009, but China’s further reduction of carbon emissions in trades is necessary due to its increasing trend of trade volumes with Germany since 2010.

Empirical estimations of this paper suggest that the causal relationship between bilateral trade volumes and embodied carbon emissions is an inverse U-shape for both of the countries, notwithstanding China’s exports to Germany having shown a stronger result both for the short-term and long-term periods and for a couple of the estimated models, such as ARDL, FMOLS, and CCR regressions. Comparatively, the above inverse U-shape pattern generated from Germany’s exports is significant for FMOLS and CCR models. Furthermore, the level of technology reduces carbon emissions embodied in trades for China. On the contrary, urbanization and China’s attending to the WTO stimulate exporting which further enhance the carbon emissions. The above results prove the importance of technology in the sustainability of trade, and higher levels of technology will lead to a lower possibility of carbon leakage and CO_2_ transfer especially for developing economies. The models in use pass various robustness tests, such as the DF-GLS test, the PP unit root test, and ARDL bound tests.

This article empirically verifies the theories which suggest nonlinear nexus between trade and the environment specifically in comparing developing countries with developed countries [9] which are both net exporting-oriented economies [15]. According to the above empirical findings, bilateral trade produced additional emissions at first, which fitted the theories of “Pollution Haven Hypothesis” [5] and “Race to the Bottom Hypothesis” [17]. In the later parts of the bilateral trades, the generated carbon emissions emit less in the long-term comparing with the increasing trade volumes, which is consistent with the theoretical view that trades enhance the environment with updates of the technology or potential technological spillover [23].

From the theoretical perspective, this paper also proposes that understanding the impacts of trade on the environment is complex but of interest. In the case of bilateral trade of China–Germany, the linkage between theoretical backgrounds and empirical analysis mainly lay in the following two aspects: the impacts of trade on the environment for developed and emerging economies reveal an inverse U-shape, nevertheless different trade structures are more likely to contribute to a large amount of net carbon emissions for less-developed countries. Therefore, a large room for the developing country to reduce carbon emissions is a main target. Second, different trade structures encourage the developing country to update technologies which improve production efficiency and finally decline the emissions. For this particular aspect, our results are consistent with Jin et al. [9]. In addition, by taking advantage of absorbing advanced technologies from the developed countries further control of carbon emissions could be possible for less developed countries. Additionally, the well-developed countries are more likely to shift technologies to a developing economy. In this case, the classic trade theory of comparative advantage [47] could be achieved in practice under the circumstances of considering the impacts of trade on the environment.

In summary, the results discover a potential theoretical contribution in linking trade and the environment for well-developed countries and emerging entities. In light of the findings, we could carefully explore trades among countries with different economic development levels and trade structures, as technological spillover possibly occurs among the above situations, and developing technology levels could contribute in constructing an inverse U-shape between trade and the environment in the long-term.

Policy implications of this article are clear. First, we confirm that the exports between China and Germany could further avoid environmental degradation in bilateral trade. Therefore, a couple of positive policies designed to stimulate bilateral trade in high value-added service productions between China and Germany could be further strengthened. Second, technology updates are significant for China to reduce CO_2_ emissions and further alleviate the carbon emission pressure. It is also important to apply renewable energy sources and improve energy efficiency in production processes at the current stage for China.

The limitations of this article are mainly of two aspects: first, the causal relationship between the trade impacts on its generated carbon emissions could reshape when China trades with other developing countries. For example, China rapidly increases trades with the Belt and Road Initiative group, and the net exports mainly concentrate on energy-intensive industries [1] and, thus, the environmental consequences from trades could be different for different trading partners and under different national strategies. Second, due to the length of this article, only the total bilateral export amount instead of each industry is estimated for empirical analysis and, hence, various industries should be explored to examine the environmental results for bilateral trade.

## Figures and Tables

**Table 1:** Definition of the main variables.

Variables	Definition
LNCO_2C,t_	Logarithm of the exporting carbon emissions from China to Germany in year t
LNCO_2G,t_	Logarithm of the exporting carbon emissions from Germany to China in year t
LNTRA_C,t_	Logarithm of the trade volume exporting from China to Germany in year t
LNTRA_G,t_	Logarithm of the trade volume exporting from China to Germany in year t
LNTRA^2^_C,t_	The squared term of LNTRA_C,t_
LNTRA^2^_G,t_	The squared term of LNTRA_G,t_
WTO_t_	China’s participation in the WTO (equals to 1 if t> = 2001; 0 otherwise)
TECH_C,t_	R&D intensity (Number of technicians per 1 million people) of China in year t
TECH_G,t_	R&D intensity (Number of technicians per 1 million people) of Germany in year t
LNURB_C,t_	Urbanization level of China in year t
LNURB_G,t_	Urbanization level of Germany in year t

**Table 2:** Descriptive statistics results for China.

Variables	LNCO_2C_	LNTRA_C_	LNTRA^2^_C_	WTO	TECH_C_	LNURB_C_
Mean	9.15	24.70	610.88	0.84	1.51	3.82
Median	9.45	24.98	624.12	1.00	1.44	3.84
Maximum	9.69	25.43	647.08	1.00	2.12	4.05
Minimum	8.05	23.30	543.27	0.00	0.74	3.55
Std. Dev.	0.59	0.75	37.01	0.37	0.44	0.16
Skewness	−1.01	−0.77	−0.75	−1.87	−0.11	−0.20
Kurtosis	2.30	2.01	1.98	4.52	1.70	1.80
J-B	3.61	2.64	2.59	12.98	1.37	1.26
*p*-values	0.16	0.26	0.27	0.00	0.50	0.53
Observations	19	19	19	19	19	19

Note: J-B represents for the Jarque–Bera test. The unit before logarithm for LNCO_2C_ is 10 kt, and the units before logarithm for LNTRA_C_ and LNTRA^2^_C_ is USD.

**Table 3:** Correlation analysis results for China.

Variables	LNCO_2C_	LNTRA_C_	LNTRA^2^_C_	WTO	TECH_C_	LNURB_C_
LNCO_2C_	1.00					
	-----					
LNTRA_C_	0.95 ***	1.00				
	(13.58)	-----				
LNTRA^2^_C_	0.95 ***	1.00 ***	1.00			
	(13.21)	(419.51)	-----			
WTO	0.79 ***	0.75 ***	0.75 ***	1.00		
	(5.31)	(4.74)	(4.67)	-----		
TECH_C_	0.79 ***	0.93 ***	0.93 ***	0.65 ***	1.00	
	(5.41)	(10.45)	(10.62)	(3.52)	-----	
LNURB_C_	0.83 ***	0.95 ***	0.95 ***	0.67 ***	0.99 ***	1.00
	(6.26)	(12.70)	(12.94)	(3.75)	(38.96)	-----

Note: t-Statistics values are in parentheses. *** denotes *p*-values at the one percent level.

**Table 4:** Descriptive statistics results for Germany.

Variables	LNCO_2G_	LNTRA_G_	LNTRA^2^_G_	WTO	TECH_G_	LNURB_G_
Mean	6.16	24.33	592.89	0.84	2.63	4.33
Median	6.35	24.58	604.43	1.00	2.59	4.33
Maximum	6.94	25.29	640.05	1.00	3.03	4.34
Minimum	4.65	22.62	512.03	0.00	2.33	4.31
Std. Dev.	0.77	0.90	43.60	0.37	0.23	0.01
Skewness	−0.76	−0.61	−0.57	−1.87	0.24	−0.51
Kurtosis	2.22	2.04	1.99	4.52	1.50	1.82
J-B	2.33	1.92	1.87	12.98	1.96	1.93
*p*-values	0.31	0.38	0.39	0.00	0.37	0.38
Observations	19	19	19	19	19	19

Note: J-B represents for the Jarque–Bera test. The unit before logarithm for LNCO_2G_ is 10 kt, and the units before logarithm for LNTRA_G_ and LNTRA^2^_G_ is USD.

**Table 5:** Correlation analysis results for Germany.

Variables	LNCO_2G_	LNTRA_G_	LNTRA^2^_G_	WTO	TECH_G_	LNURB_G_
LNCO_2G_	1.00					
	-----					
LNTRA_G_	0.99 ***	1.00				
	(39.89)	-----				
LNTRA^2^_G_	0.99 ***	0.99 ***	1.00			
	(36.09)	(276.69)	-----			
WTO	0.78 ***	0.75 ***	0.74 ***	1.00		
	(5.17)	(4.74)	(4.62)	-----		
TECH_G_	0.85 ***	0.89 ***	0.89 ***	0.50 **	1.00	
	(6.74)	(8.15)	(8.38)	(2.39)	-----	
LNURB_G_	0.98 ***	0.99 ***	0.99 ***	0.71 ***	0.91 ***	1.00
	(24.78)	(39.92)	(41.86)	(4.26)	(9.25)	-----

Note: t-Statistics values are in parentheses. *** and ** denote *p*-values at the one and five percent levels, respectively.

**Table 6:** The bilateral trades amounts and their embodied carbon emissions from 1999 to 2018.

Year	China’s Carbon Emissions	Germany’s Carbon Emissions	Net Carbon Emissions of China	China’s Total Exports	Germany’s Total Exports	Net Exports of China
1999	3144.16	105.03	3039.13	13.26	6.72	6.54
2000	3400.34	115.87	3284.47	15.22	7.61	7.61
2001	3386.39	147.65	3238.74	16.14	9.83	6.31
2002	3784.67	183.83	3600.83	18.61	12.70	5.91
2003	5441.71	283.07	5158.63	25.80	19.26	6.55
2004	8852.72	361.18	8491.54	37.83	24.77	13.07
2005	10,981.69	361.61	10,620.08	47.19	25.61	21.58
2006	13,509.55	487.27	13,022.28	59.29	32.81	26.48
2007	15,882.54	542.34	15,340.20	69.49	38.96	30.53
2008	16,290.40	574.15	15,716.25	79.95	47.56	32.39
2009	12,397.55	678.98	11,718.57	70.76	48.68	22.08
2010	14,841.05	871.28	13,969.77	96.02	68.52	27.50
2011	14,617.50	1040.85	13,576.65	106.57	87.71	18.86
2012	12,511.87	971.43	11,540.43	97.74	84.23	13.52
2013	13,063.44	982.30	12,081.14	96.03	87.53	8.50
2014	13,188.49	963.56	12,224.92	103.06	97.10	5.96
2015	12,712.40	795.41	11,916.99	98.96	78.05	20.91
2016	12,954.31	852.03	12,102.28	101.13	83.39	17.73
2017	14,274.37	975.69	13,298.68	111.57	95.29	16.28
2018	15,678.97	1095.36	14,583.60	121.45	108.47	12.98

Note: Column (2) to Column (4) are embodied carbon emissions generated from the trade amounts with the unit of 10 kt. The last three columns are the trade amounts with the unit of 1 billion USD.

**Table 7:** Unit root tests results for Model 1—Bilateral trade from China to Germany.

DF-GLS Test Statistic			PP Test Statistic	
Series	Level	First Difference	Level	First Difference
LNCO_2C_	−2.58	−3.10 *	−1.32	−3.03 *
LNTRA_C_	−1.34	−3.65 **	−0.61	−3.55 *
LNTRA^2^_C_	−1.33	−3.69 **	−0.61	−3.62 **
TECH_C_	−2.23	−3.93 ***	−1.87	−4.86 ***
WTO	−2.10	−4.86 ***	−2.50	−4.72 ***
LNURB_C_	−1.49	−4.31 ***	1.10	−8.92 ***

Note: ***, **, and * denote the significance levels at 1%, 5%, and 10%, respectively.

**Table 8:** Unit root tests results for Model 2—Bilateral trade from Germany to China.

DF-GLS Test Statistic			PP Test Statistic	
Series	Level	First Difference	Level	First Difference
LNCO_2G_	−1.72	−3.97 ***	−0.43	−6.57 ***
LNTRA_G_	−0.96	−4.55 ***	0.07	−8.92 ***
LNTRA^2^_G_	−0.98	−4.53 ***	0.00	−8.61 ***
TECH_G_	−3.33	−3.47 **	−1.86	−4.13 **
WTO	−2.10	−4.86 ***	−2.50	−4.72 ***
LNURB_G_	−2.03	−4.27 ***	−2.13	−3.61 **

Note: t-Statistics values are in parentheses. *** and ** denote *p*-values at the one and five percent levels, respectively.

**Table 9:** ARDL bounds cointegration testing results.

	Model 1		Model 2	
Test Statistic	Value		Test Statistic	Value
K = 5	10.62 ***		K = 5	3.90 *
Significance	I0 Bound	I1 Bound		
10%	2.75	3.79		
5%	3.12	4.25		
2.5%	3.49	4.67		
1%	3.93	5.23		
Diagnostic tests				
R^2^	0.99		0.95	
Adj-R^2^	0.98		0.88	
F-statistic	114.38		14.56	
Prob(F-statistic)	0.00		0.00	

Note: *** and * denote the significance levels at 1% and 10%, respectively.

**Table 10:** Long-run and short-run results of the ARDL method for Model 1.

Variables	Coefficient	Std. Error	t-Statistic	*p*-Values
Long-run analysis				
LNTRA_C_	22.19 ***	1.12	19.70	0.00
LNTRA^2^_C_	−0.43 ***	0.02	−18.71	0.00
TECH_C_	−1.19 ***	0.08	−14.18	0.00
WTO	0.06 **	0.02	2.69	0.03
LNURB_C_	1.59 ***	0.28	5.63	0.00
Constant	−275.52 ***	13.24	−20.79	0.00
Short-run analysis				
∆LNCO_2C_ (−1)	−0.51 **	0.19	−2.69	0.03
∆LNTRA_C_	3.25	2.47	1.31	0.22
∆LNTRA_C_ (−1)	30.34 ***	5.26	5.76	0.00
∆LNTRA^2^_C_	−0.05	0.04	−1.14	0.29
∆LNTRA^2^_C_(−1)	−0.60 ***	0.10	−5.86	0.00
∆TECH_C_	−1.32 ***	0.16	−8.167	0.00
∆TECH_C_ (−1)	−0.49 ***	0.21	−2.25	0.05
∆WTO	0.10 **	0.03	2.68	0.03
∆LNURB_C_	−46.82 **	6.05	−7.72	0.00
∆LNURB_C_ (−1)	49.23 ***	6.22	7.91	0.00
ECM(−1)	−1.51 ***	0.19	−7.92	0.00
Diagnostic tests				
R^2^	0.99			
Adj-R^2^	0.99			
F-statistic	254.81			
Prob(F-statistic)	0.00			
Normality test	0.79 (0.67)			
Serial Correlation	1.49 (0.26)			
ARCH	1.97 (0.17)			
Ramsey	0.31 (0.76)			
CUSUM	Stable			
CUSUMSQ	Stable			

Note: *** and ** denote the significance levels at 1% and 5%, respectively.

**Table 11:** Robustness analysis for Model 1.

	FMLOS	CCR
Variables	Coefficient	Coefficient
LNTRA_C_	14.79 (0.00)	14.29 (0.00)
LNTRA^2^_C_	−0.29 (0.00)	−0.27 (0.00)
TECH_C_	−1.40 (0.00)	−1.43 (0.00)
WTO	−0.13 (0.02)	−0.11 (0.19)
LNURB_C_	2.68 (0.00)	2.74 (0.00)
Constant	−192.17 (0.00)	−186.21 (0.00)
R^2^	0.99	0.99
Adj-R^2^	0.99	0.99

Note: Numbers in parentheses are *p*-values.

**Table 12:** Long-run and short-run results of the ARDL method for Model 2.

Variables	Coefficient	Std. Error	t-Statistic	*p*-Values
Long-run analysis				
LNTRA_G_	5.08	3.26	1.55	0.16
LNTRA^2^_G_	−0.08	0.06	−1.28	0.24
WTO	−0.04	0.07	−0.65	0.53
TECH_G_	0.30	0.45	0.66	0.52
LNURB_G_	11.82	12.07	0.97	0.36
Constant	−117.19 *	60.47	−1.93	0.09
Short-run analysis				
∆LNCO_2G_(−1)	−0.32	0.57	−0.56	0.58
∆LNTRA_G_	0.54	4.68	0.11	0.91
∆LNTRA_G_(−1)	6.19	4.51	1.37	0.21
∆LNTRA^2^_G_	0.00	0.09	0.06	0.94
∆LNTRA^2^_G_(−1)	−0.12	0.08	−1.40	0.20
∆WTO	−0.06	0.08	−0.69	0.51
∆TECH_G_	−0.00	0.42	−0.00	0.99
∆TECH_G_(−1)	0.40	0.29	1.38	0.20
∆LNURB_G_	15.66	16.57	0.94	0.37
ECM(−1)	−1.32 **	0.57	−2.306	0.05
Diagnostic tests				
R^2^	0.99			
Adj-R^2^	0.99			
F-statistic	350.88			
Prob(F-statistic)	0.00			
Normality test	0.21(0.89)			
Serial Correlation	1.73 (0.26)			
ARCH	0.89 (0.35)			
Ramsey	0.11 (0.91)			
CUSUM	Stable			
CUSUMSQ	Stable			

Note: ** and * denote the significance levels at 5% and 10%, respectively.

**Table 13:** Robustness analysis for Model 2.

	FMLOS	CCR
Variables	Coefficient	Coefficient
LNTRA_G_	3.98 (0.00)	4.96 (0.03)
LNTRA^2^_G_	−0.06 (0.03)	−0.08 (0.06)
WTO	−0.052 (0.29)	−0.09 (0.34)
TECH_G_	0.084 (0.59)	0.18 (0.40)
LNURB_G_	5.60 (0.43)	−4.58 (0.72)
Constant	−77.67 (0.01)	−46.93 (0.27)
R^2^	0.99	0.99
Adj-R^2^	0.99	0.99

Note: Numbers in parentheses are *p*-values.
